# Application of heterogeneous catalysts in the preparation of bio-based platform compound 5-hydroxymethylfurfural

**DOI:** 10.1186/s40643-025-00894-5

**Published:** 2025-06-06

**Authors:** Yuyuan He, Jinrong Zhou, Han Li, Lin Deng, Jinnan Gao

**Affiliations:** 1 Luoyang Institute of Science and Technology, Luoyang, 471023 China; 2 College of Animal Science and Technology, Henan Agricultural University, Zhengzhou, 450046 China; 3 College of Life Sciences, Inner Mongolia Agricultural University, Hohhot, 010018 China

**Keywords:** 5-Hydroxymethylfurfural, Platform compound, Catalytic conversion, Heterogeneous catalyst

## Abstract

In recent years, there has been growing attention towards developing renewable energy and materials derived from abundant biomass resources. 5-Hydroxymethylfurfural (HMF) is recognized as a promising bio-based platform compound for synthesizing value-added chemicals and materials due to its versatile reactivity. HMF can be directly synthesized from carbohydrates and various raw biomass through the acid-hydrolysis reaction. Heterogeneous catalysts have gained prominence in biomass conversion owing to their environmental friendliness, facile separation from reaction mixtures, high catalytic efficiency, and reduced corrosivity toward equipment. This review systematically examines the reaction pathways and mechanisms involved in HMF synthesis from fructose, glucose, cellulose, and raw biomass using heterogeneous catalysts. Then we give an introduction to the preparation of furandicarboxylic acid (FDCA) from HMF with different catalytic methods. FDCA is an important degradable bio-material monomer for polyethylene furanoate to replace petroleum-based polyethylene terephthalate. This review ends with a prospect on the challenges and opportunities of HMF synthesis in the near future.

**Graphical abstract:**

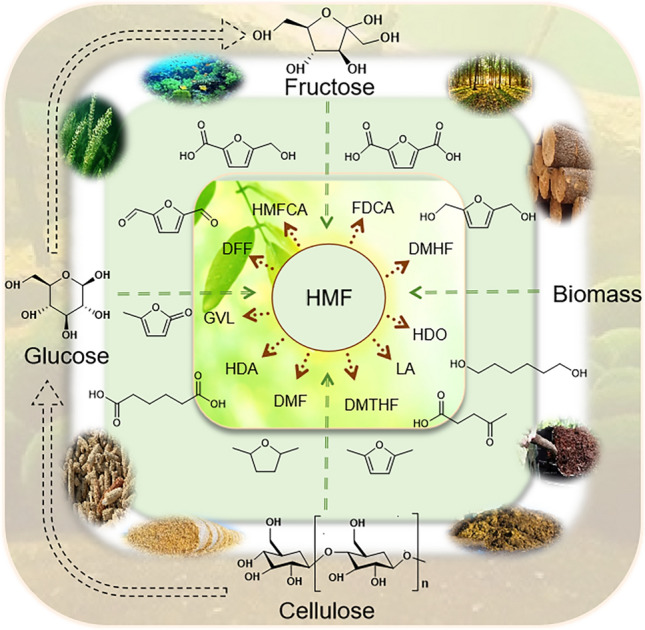

## Introduction

With the excessive fossil energy consumption, more and more attention has been paid to the development of renewable energy sources. Biomass is the only renewable carbon resource that has great potential to replace fossil resources (Švarc-Gajić et al. 2022; Román-Leshkov et al. 2007). In 2004, the US Department of Energy selected 12 high-value platform chemicals among over 300 compounds derived from biomass (Xia et al. 2022). 5-Hydroxymethylfurfural (HMF), a promising bio-based platform compound, exhibits high reactivity, making it a versatile precursor for synthesizing renewable chemicals. It is predominantly derived from the acid-catalyzed dehydration of hexoses (e.g., fructose, glucose), which are abundant in carbohydrate-rich biomass feedstocks such as lignocellulosic materials, agricultural residues, and algal polysaccharides (Chen and Ge 2025; Cunha et al. 2025). HMF can be directly prepared from monosaccharides, disaccharides and raw biomass under mild conditions through one-pot reactions, including hydrolysis, isomerization, and dehydration (Du et al. 2023; Takagaki et al. 2009). HMF is known as the “sleeping giant” in the field of new materials and has been the focus of academic and industry attention (Kang et al. 2025). A series of HMF derivatives ‌is‌ produced through oxidation, hydrogenation, esterification, and other reactions, enabling the synthesis of ‌end products‌ for applications in industries such as plastics manufacturing, food additives, and specialty chemicals (Wang et al. 2025a). Furandicarboxylic acid (FDCA) from HMF oxidation can be used as a substitute for the petrochemical derivative terephthalic acid, which has wide application prospects (Long et al. 2025).

In recent years, much progress has been made in HMF synthesis from various biomass feedstocks with different homogeneous and heterogeneous acid catalysts (Niakan et al. 2025; Wang et al. 2025c). Compared with homogeneous catalysts, heterogeneous catalysts exhibit a key advantage: ‌they can be easily separated from the reaction mixture post-synthesis and reused‌, significantly reducing operational costs (Kim et al. 2025; Wang et al. 2025b). Herein, the advances in synthesizing HMF catalyzed by heterogeneous catalysts in recent years were summarized. These findings in HMF synthesis were classified by various feedstock including fructose, glucose, cellulose, and raw biomass via acid-catalyzed conversion. Then, the preparation of FDCA derived from HMF was introduced briefly. Finally, the challenges and perspectives for HMF synthesis with heterogeneous catalysts were discussed. 

## The synthesis of HMF

The primary sources of raw materials for producing HMF are biomass and agricultural waste rich in sugars (De Andrades et al. 2024). This chapter mainly reviewed the preparation of HMF from glucose, fructose, cellulose, and raw biomass as feedstock with various catalysts. While preparing HMF, the solvent systems used for the reaction contained single-phase and biphasic systems and the catalysts can be divided into homogeneous and heterogeneous catalysts. Homogeneous catalysts typically include inorganic acids, organic acids, ionic liquids, and some metal salts, while heterogeneous catalysts mainly include solid acids, solid bases, and other solid catalysts.

## Homogeneous catalysts

Currently, the main homogeneous catalysts used for preparing HMF are inorganic acids, organic acids, metal salts, and ionic liquids. The key advantage of homogeneous acid catalysts lies in their ‌uniform dispersion of active sites within the solvent‌, ensuring ‌full contact with the reaction substrate‌ and thereby enhancing catalytic efficiency (Niakan et al. 2025; Thiensuwan et al. 2025). This type of catalyst exhibits high catalytic activity and selectivity. In contrast, homogeneous acids suffer from significant drawbacks, such as difficulty in recovery, excessive wastewater generation, and high operational costs, which fail to align with the principles of environmentally friendly chemistry(Rostami et al. 2025). At the same time, researchers also explored the effect of different solvent systems on HMF production. The solvent systems currently reported are mainly water or mixed solvents of water with ionic liquids and organic solvents. The mixed solvents usually can form biphasic systems, and the organic phase can protect HMF and suppress unnecessary side reactions during the reaction process.

### Inorganic acids

**Fig. 1:**
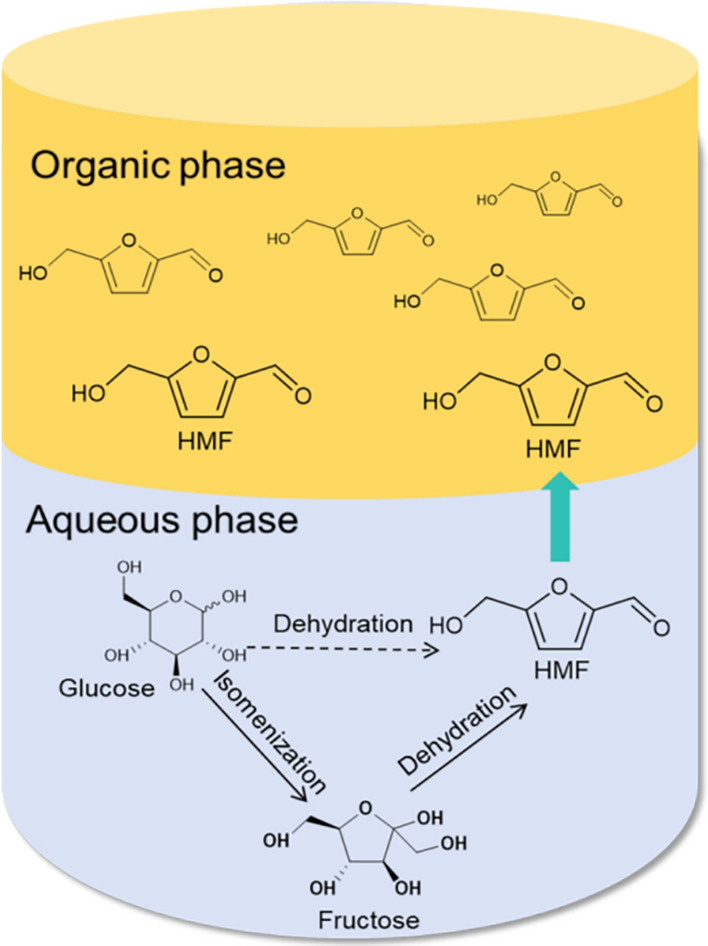
HMF synthesis in a biphasic system

Inorganic acid catalysts (HCl, H_2_SO_4,_ HNO_3,_ and H_3_PO_4_) are mainly Bronsted acids for HMF synthesis from fructose and glucose (De Andrades et al. 2024). Román-Leshkov et al. employed inorganic acids (HCl, H_2_SO_4,_ and H_3_PO_4_) as the catalyst to synthesize HMF from fructose in the biphasic phase system (Román-Leshkov et al. 2006a). HMF yield was only 25.5% in H_2_O and 54.6% in H_2_O/methyl isobutyl ketone (H_2_O/MIBK) at 180 °C. Then, dimethyl sulfoxide (DMSO) was introduced to the mixed solvent and a 62.3% yield of HMF was obtained. Compared with single-phase solvent, the yield of HMF synthesis in biphasic solvent is higher. As shown in Fig. 1, glucose was converted into HMF in the aqueous phase, and the organic phase can simultaneously extract HMF from the aqueous phase and avoid its side reactions. Svenningsen et al. converted fructose dehydration to HMF using inorganic acids (HI, HBr, HCl, H_2_SO_4_, and HNO_3_) at 120 °C and found that the selectivity to HMF from fructose reached up to ~ 83% (Svenningsen et al. 2018). They also studied the selectivity of HMF in different solvent environments. DMSO, N-methyl pyrrolidone, dimethylacetamide, and N, N-dimethylformamide were mixed with H_2_O as solvents for fructose conversion at 10 °C for 10 min, and 84.0%, 77.0%, 78.0%, and 74.0% of HMF selectivity were obtained, respectively. From the above research, it can be seen that the introduction of the organic phase can contribute to HMF synthesis from glucose and fructose.

### Organic acids

Compared with common inorganic acids, organic acids can form a milder reaction environment, while their acidity is weaker. Sajid et al. used p-toluenesulfonic acid (pTSA) and oxalic acid as catalysts for converting fructose to HMF (Sajid et al. 2020). When pTSA acted as the catalyst in the DMSO medium, 90.2% of HMF yield can be reached at 120 °C for 30 min. 84.1% of HMF yield was achieved at 130 °C for 2 h with oxalic acid. Yu et al. used rice waste as raw material to prepare HMF (Yu et al. 2019). The reaction was carried out in H_2_O/DMSO solvent at 140 °C for 100 min, and HMF yields obtained were 35.2 mol% and 17.3 mol% using Al(III) and Al/Maleic acid as catalysts, respectively. The Lewis acidity of Al(III) was regulated through the appropriate coordination of maleic acid-Al(III). This coordination inhibited the side reactions and facilitated HMF formation. Therefore, the catalytic ability of organic acids is not as good as that of inorganic acids, which are usually used in combination with other catalysts to achieve excellent catalytic effects.

### Metal salts

**Fig. 2:**
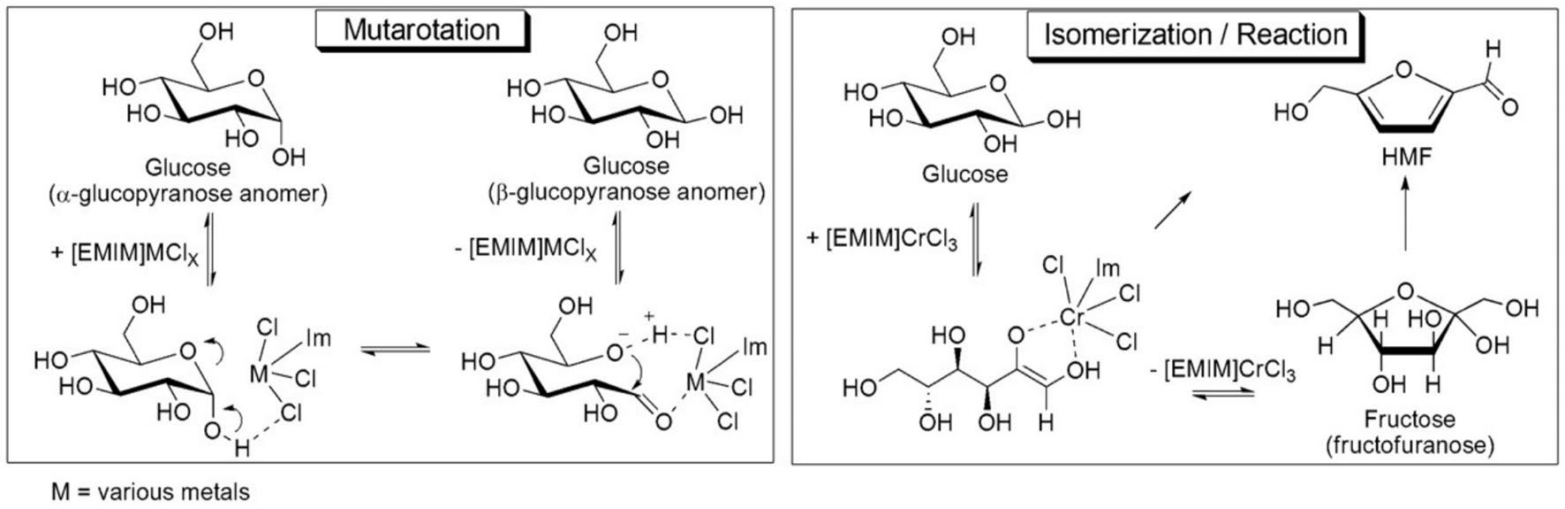
Proposed metal halide interaction with glucose and HMF synthesis in [EMIM]Cl (Zhao et al. 2007)

Metal salts dissolving in solution forming usually contain Lewis and Bronsted acid centers and have the advantage of high efficiency, good selectivity, and mild reaction conditions. Zhao et al. prepared HMF from glucose catalyzed by metal halide (Zhao et al. 2007). They found that the yield of HMF catalyzed by H_2_SO_4_ or AlCl_3_ in [EMIM]Cl was only 10.0% at 100 °C. Surprisingly, CrCl_2_ provided 70.0% of HMF yield. This is due to that glucose starting material is predominantly an α-anomer dissolved in [EMIM]Cl, and equilibrium of a mixture of anomers caused by mutarotation (α- to β-anomer conversion) was rapidly formed in CuCl_2_ or CrCl_2_. The proposed metal halide interaction with glucose and HMF synthesis is shown in Fig. 2. Shi et al. employed NaHSO_4_-ZnSO_4_ as a cocatalyst and obtained a yield of 53.0% of HMF from cellulose in H_2_O/THF medium at 160 °C for 60 min (Shi et al. 2013). Yan et al. converted cellulose into HMF directly using RuCl_3_ as the catalyst and achieved 83.3% HMF yield in the NaCl aqueous solution/butanol biphasic system (Yan et al. 2019). Yu et al. employed cooked rice and bread as raw materials catalyzed by SnCl_4_ and obtained HMF and glucose yields of 8.1–9.5% and 44.2–64.8%, respectively (Yu et al. 2016). HMF yields of 69.2 ± 4.8%, 62.8 ± 1.9%, and 60.4 ± 9.3% from fructose were obtained with FeCl_3_, SnCl_4_, and CuCl_2_ as catalysts. Although metal salt catalysts exhibited excellent catalytic performance, they were still limited by the difficulty in recovery and product separation.

### Ionic liquids

**Fig. 3:**
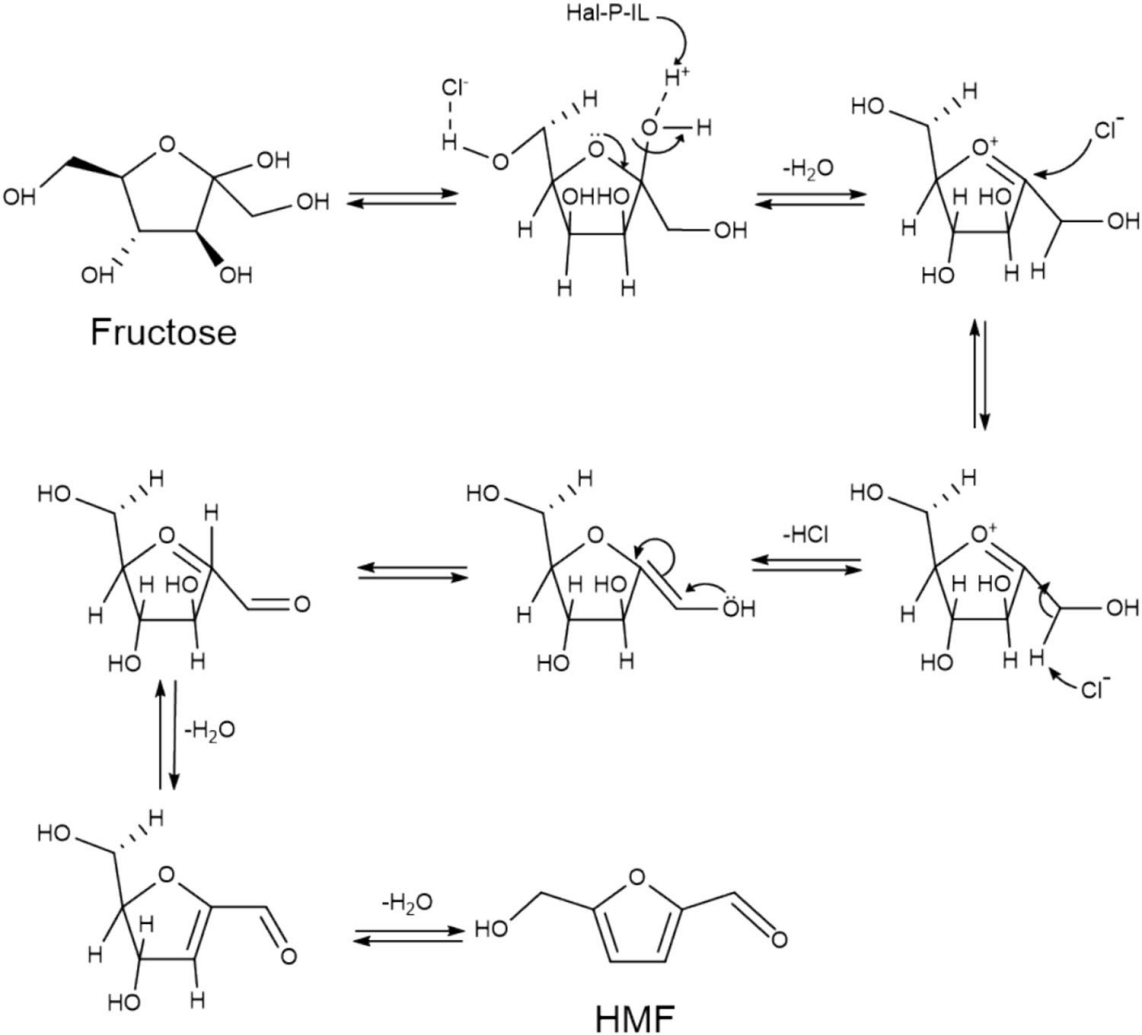
Reaction mechanism of fructose conversion to HMF catalyzed by Hal-P-IL (Sadjadi et al. 2023)

Ramli et al. employed [SMIM][FeCl_4_] to convert glucose to HMF with an 18.0% yield, and the catalyst was reused for five cycles without significant loss in catalytic activity (Ramli and Amin 2015). Tao et al. studied the HMF synthesis from fructose catalyzed by ionic liquid 1-(4-sulfonic acid) butyl-3-methylimidazole hydrogen sulfate in H_2_O/MIBK at 120 °C for 180 min, achieving 100% fructose conversion and 94.6%HMF yield, the ionic liquid 1-(4-sulfonic acid) butyl-3-methylimidazole hydrogen sulfate could be recycled and exhibited constant activity for six successful runs (Tao et al. 2011). Hu et al. prepared choline-based ionic liquids with cheap citric acid and choline chloride, and the highest HMF yield from fructose was 91.4% in the ionic liquid/ethyl acetate biphasic system. However, after repeating the run 8 times, the yield decreased slightly. This was due to the increase of water content in the ionic liquids because the reaction produced water (Hu et al. 2008). Moreau et al. used ionic liquid 3-methylimidazole chloride as a catalyst for preparing HMF from fructose as raw material and obtained the 92.0% HMF yield at 90 °C for 45 min, the yield of HMF decreased to 62% after the ionic liquid was recycled five times (Moreau et al. 2006). Sadjadi et al. employed polyionic liquids to regulate the halloysite acidity and prepared acidic ionic liquid catalysts (Hal-P-IL) to convert fructose to HMF. For other runs, only slight a loss of the catalytic activity of the catalyst was observed, and HMF yield decreased to 83.2% after seven runs (Sadjadi et al. 2023). The maximum HMF yield was 97.8% in H_2_O/DMSO medium with 20 wt% loading of Hal-PIL catalyst at 70 °C for 30 min. The mechanism of HMF synthesis from fructose in the presence of Hal-PIL is shown in Fig. 3. Ionic liquids have a dual function as both catalysts and solvents and have the characteristics of high catalytic efficiency and high reuse. However, the high cost of ionic liquids limits their wide application as catalysts and solvents.

## The synthesis of HMF from carbohydrates and biomass feedstocks

### The synthesis of HMF from fructose

Solid acid catalysts consist of active centers that are both Bronsted acids and Lewis acids. Solid acid catalysts have advantages such as easy recovery and separation, high specific surface area, and low corrosion to equipment, and have been increasingly studied in recent years (Bi et al. 2025; Svenningsen et al. 2018).

**Fig. 4:**
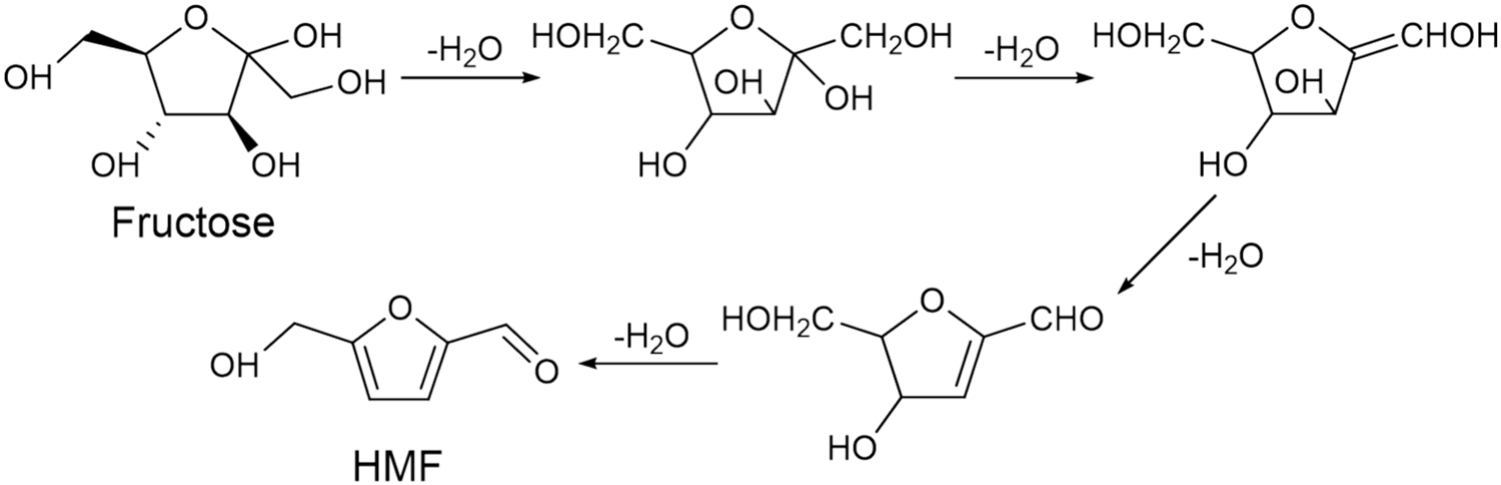
Reaction mechanism of fructose dehydration to prepare HMF (Kuster 1990)

Fructose has abundant sources which can be widely found in fruits and vegetables, and offers a more practical and promising approach for producing HMF(Aranha et al. 2025; Dang et al. 2025). Fructose can be easily dehydrated to form HMF under mild reaction conditions (Román-Leshkov et al. 2007). In contrast, most other carbohydrates, such as glucose, starch, and cellulose, mainly require prior fructose degradation before converting into HMF. Solid acid catalysts have advantages such as easy recovery and separation, high specific surface area, and low corrosion to equipment. Recently, many novel solid acid catalysts have been developed, mainly including metal oxides, halogen-free solid superacids, ion-exchange resins, heteropolyacids (HPAs), zeolites, and bio-based carbon materials. Ion-exchange resins are generally suitable for catalytic reactions below 130 °C and are not eligible for high-temperature catalytic biomass conversion due to their inherent organic structure. Bio-based carbon materials have some drawbacks, such as high energy consumption for high-temperature carbonization, a large amount of concentrated sulfuric acid or fuming sulfuric acid required for sulfonation, and low acid density. This section summarized the effect of various catalysts on fructose conversion. The reaction mechanism of fructose dehydration to prepare HMF is shown in Fig. 4.

Metal oxides have high catalytic activity and were used in the catalytic conversion of biomass. Zhong et al.studied the catalytic activity of protonation and layered tantalum-based oxides in HMF synthesis from fructose (Zhong et al. 2017). 67% HMF yield is achieved in DMSO using layered HTaWO_6_. Zhao et al. prepared TiZrO metal oxide catalyst using isopropyl titanate and zirconium n-butanol as precursors and introduced phosphorus to enhance the acidity of the catalyst (TiZrPO), (Zhao et al. 2020a, b). They found TiZrPO-2 exhibited the most superior catalytic performance, with 98.5% fructose conversion and 76.5% HMF yield at 170 °C for 40 min. The catalyst can provide a suitable specific surface area, increase acid center density and acid, and improve mechanical strength by modifying the catalyst carrier. Even after four recycling cycles, the catalyst TiZrPO-2 maintained a fructose conversion rate of 92.7% and an HMF yield of 72.6%, demonstrating excellent reusability performance. García-López et al. employed bare Nb_2_O_5_, Nb_2_O_5_-TiO_2_, or Nb_2_O_5_-CeO_2_ to prepare HMF from fructose in the aqueous medium, and the highest yield of 57% was achieved using pristine Nb_2_O_5_ at 165 °C for 40 min (García-López et al. 2021). Costa et al. converted fructose into HMF using NbOPO_4_ as the catalyst assisted by microwave heating in the biphasic system (saturated NaCl solution and MIBK), and 53% HMF yield of was obtained (Costa et al. 2023). Compared with NbOPO_4_ as the catalyst, Nb-MG (Nb(V) oxide grafted on the mesoporous glass surface), converted fructose dehydration with higher HMF productivity due to the high dispersion of Nb(V) species on the surface.

The acidity of solid superacids is stronger than that of 100% sulfuric acid. Solid superacids with high acidity are usually prepared through sulfation (–HSO_3_) and sulfonation (SO_4_^2−^) on the supports or metal oxide, which offers the advantage of excellent thermal stability, and remarkable catalytic activity. Testa et al. synthesized TiO_2_-SO_3_H through sulfonating TiO_2_ support, which was used to produce HMF from fructose, and the 71% HMF selectivity was achieved with a lower fructose concentration (0.1 M) at 140 °C (Testa et al. 2020). Niakan et al. grafted mercaptopropyl groups to SBA-16 with propylsulfonic acid, and then formed sulfonic acid groups with H_2_O_2_ oxidation. The prepared SO_3_H@SBA-16 was used to produce HMF from fructose, and the 91% HMF yield was reached in H_2_O/DMSO medium at 110 °C for 60 min. After the six-run recycling test, SO_3_H@SBA-16 still keeps a high catalytic performance. Qi et al. prepared SO_4_^2−^/ZrO_2_ through H_2_SO_4_ impregnation, and converted fructose to HMF through microwave heating in acetone/DMSO mixtures at 180 °C for 20 min, achieving 93.6% fructose conversion and 72.8% HMF yield (Qi et al. 2009a). Compared to ZrO_2_ as the catalyst, SO_4_^2−^/ZrO_2_ catalyzed fructose to form HMF with a dramatically improved yield. Tomer and Biswas prepared SO_4_^2−^/TiO_2_ catalysts with different Lewis and Brønsted site proportions by adjusting the concentration of SO_4_^2−^ (Tomer and Biswas 2020). They found that HMF yield was 74.7% with fructose as the feedstock using 0.5 M SO_4_^2−^/TiO_2_ at 150 °C for 6 h. It was perceived that the activity as well as the yield of 5-HMF reduced in each cycle and after cycle-3, glucose conversion, and 5-HMF yield reduced to 67.1% and 20.7%, respectively.

**Fig. 5:**
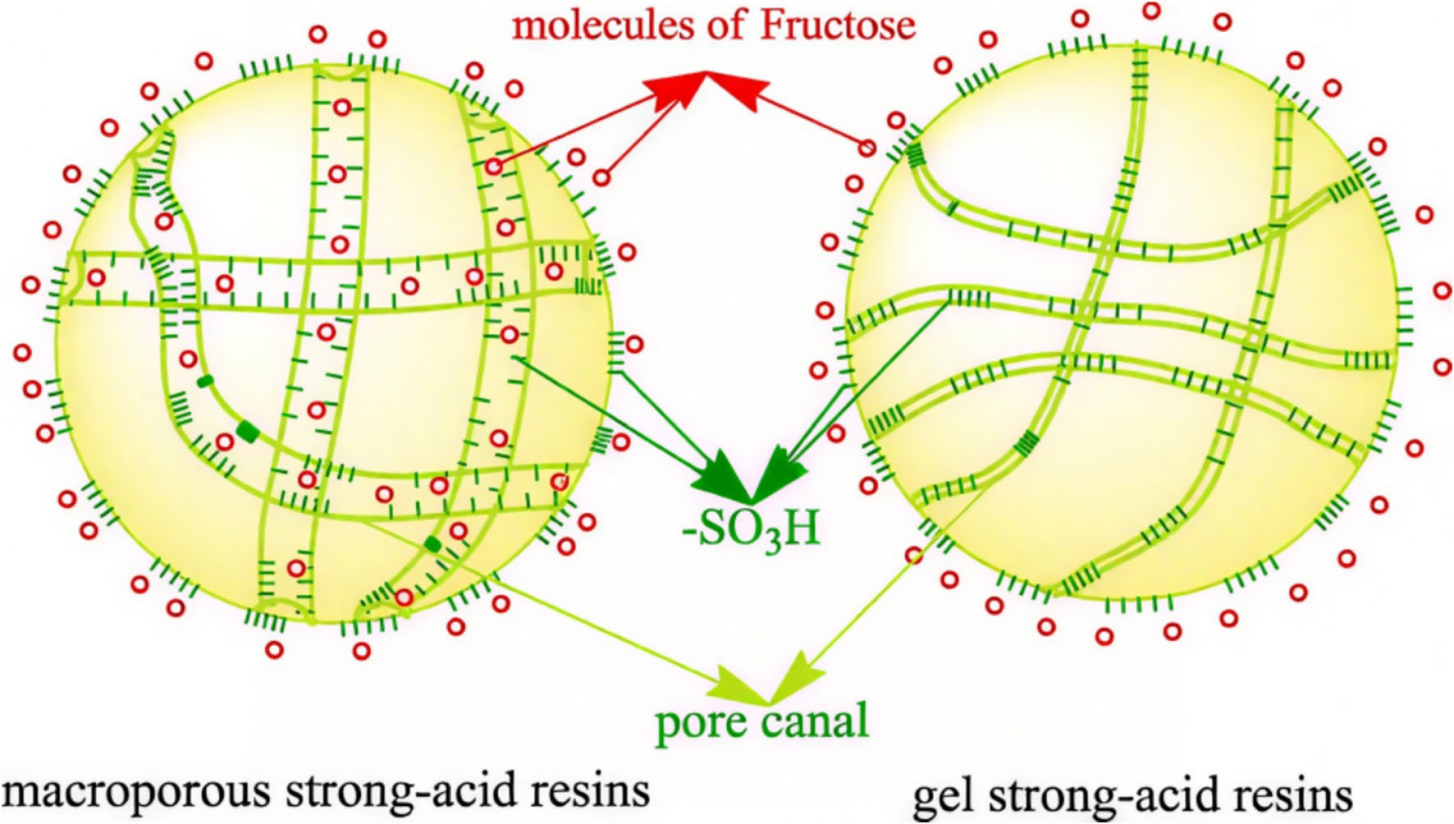
The schematic of the interaction between ion-exchange resins and fructose (Li et al. 2013)

Ion-exchange resins are the copolymers of butadiene and styrene, which are prepared as acid functional catalysts by introducing sulfonic acid. The schematic of the interaction between ion-exchange resins and fructose is shown in Fig. 5 (Li et al. 2013). Qi et al. synthesized HMF from fructose catalyzed by Amberlyst-15 in [BMIM][Cl] mixed with various common solvents at room temperature (Qi et al. 2009b). The yield of HMF was 78.0–82.0% in common solvents, including acetone, DMSO, methanol, ethanol, and ethyl acetate at 25 °C for 6 h. They also found that the HMF yield obtained with supercritical CO_2_ was similar to that of common solvents. Morales et al. employed Amberlyst-70 to prepare HMF from fructose in the H_2_O/DMSO medium, and a 93% HMF yield was obtained. The catalyst can be reused once without appreciable loss of activity, but subsequent reuse leads to a gradual decrease in both glucose conversion and HMF yield. This is probably due to the formation of organic matter deposits on the catalytic centers of Amberlyst-70 that cannot be removed by dual washing with n-hexane and methanol. These deposits limit the accessibility of new reactant molecules to the sulfonic acid sites (Morales et al. 2014). Román-Leshkov et al. investigated ion-exchange resin to catalyze fructose to HMF in biphasic systems (Román-Leshkov et al. 2006a). When H_2_O/dimethyl phenyl ether was the aqueous phase and MIBK/butanol was the organic phase, the yield of HMF from fructose was 50.32%. When H_2_O/DMSO/PVP [(poly(1-vinyl-2-pyrrolidinone)] was the aqueous phase and MIBK was the organic phase, 48.3% HMF yield can be achieved. Wu et al. employed a mild catalytic strategy for HMF synthesis from fructose catalyzed by Amberlyst-15 in H_2_O/MIBK biphasic system (Wu et al. 2024). The 46.6% HMF yield can be achieved at 90 °C for 7 h and 99.0% high-purity HMF was obtained through simple purification. Therefore, when fresh Amberlyst-15 (10 wt%) was added to the system during the third recycling run, the HMF yield became comparable to that obtained in the initial catalytic cycle.

HPAs were widely used in catalytic reactions because of their strong Bronsted acidity, high proton mobility, and strong electron conductivity (Nayak et al. 2020). However, the reasons limiting the application of HPAs mainly are their low specific surface area and high difficulty separation from reaction mixtures. Researchers have tried to synthesize the insoluble heteropolyacid-based catalysts through loading on insoluble support, substituting H^+^ in HPAs, or other approaches. HPAs loaded on the supports can also improve their specific surface area. Song et al. prepared a serial heteropolyacid salts by substitution of H + in phosphotungstic acid (H_3_PW_12_O_40_, HPW), and the highest HMF yield from fructose was 99.40% by using CePW_12_O_40_ in sec-butanol (Song et al. 2016). The HMF yield can still be 93.3% after six cycles with CePW_12_O_40_ as the catalyst. Zhao et al. prepared several bifunctional HPAs Ly_3-x_H_x_PW_12_O_40_ (Ly, lysine) by varying the ratios of HPW and lysine (Zhao et al. 2014). Ly_2_HPW_12_O_40_ achieved 92.3%HMF yield from fructose within 1 min using choline chloride as a solvent at 110 °C in preparing HMF from fructose dehydration. Ly_2_HPW showed good catalytic performance due to the synergistic effect between specific acidic and alkaline properties. Saghandali et al. prepared a highly active catalyst silicotungstic acid supported on halloysite nanoclay to produce HMF from fructose in DMSO solvent (Saghandali et al. 2024). The best reaction conditions for HMF synthesis were obtained by Response Surface Methodology, and the yield was 99.5% at 125 °C for 43 min. The HMF yield decreased to 87% after five successive catalytic cycles, indicating a 13% reduction in catalytic efficiency upon the fifth reuse.

Zeolites are silicate and aluminate compounds with regular pore structures and adjustable pore size, which can adsorb and transfer molecules selectively. Due to the unique pore structure and high specific surface area of zeolites, they have the characteristics of high efficiency, environmental protection, and controllability. Moreau et al. employed H-mordenite zeolite to prepare HMF from fructose dehydration in the H_2_O/MIBK system, and the HMF yield can reach 74% at 165 ^o^C for 90 min (Moreau et al. 1996). Jia et al. investigated the structure-performance relationships of various zeolites for HMF synthesis from fructose with microwave-assisted in H_2_O/organic solvent systems (Jia et al. 2019). They used various zeolites (MFI, BEA, and Y) as the catalysts for HMF synthesis, and achieved 72.4% fructose conversion and 49.2% HMF yield at 160 °C and 20 min. This can be due to the synergistic effect of interactions among the substrate, catalyst, and solvent. Xiang et al. employed a commercial hierarchical zeolite catalyst (HY-30) for HMF synthesis from fructose with microwave-assisted in H_2_O/DMSO/MIBK/2-BuOH biphasic medium, and 73.9% HMF yield was achieved at 160 °C for 45 min (Xiang et al. 2023). Zeolites were used for catalytic reactions at high temperatures, which was beneficial for the formation of HMF compared to its decomposition. After an initial decrease of approximately 8% in selectivity and 10% in HMF yield following the first catalytic cycle, the performance remained stable thereafter until the fifth cycle (run 5), which exhibited a further 5% decline in product yield while maintaining constant selectivity.

Functional carbon-based catalysts were prepared by using cheap, easily available, and renewable biomass in recent years. Carbon-based catalysts exhibited good hydrophobicity, high catalytic efficiency, stability, renewability, etc. Wang et al. synthesized a novel sulfonation carbon-based material (Glu-TsOH) with –SO_3_H, –OH, –COOH groups through one-step synthesis from glucose and *p*-toluenesulfonic acid (Wang et al. 2011). Then, it was employed for HMF synthesis from fructose, and HMF yield was 91.2% at 130 °C for 1.5 h. This good performance was due to the synergic effect between carboxylic acid and sulfonic acid groups. The catalyst also displayed a good reusability. Even after five cycles, the conversion and selectivity still retain 99.9% and 89.1%, respectively. Zhang et al. synthesized the carbon-based catalyst through the sulfonation of chitosan with sulfanilic acid and isoamyl nitrite (Zhang et al. 2020a). The HMF yield from fructose using 4 KSCC as the catalyst in H_2_O/organic solvent [THF, 1,4-Dioxane, γ-butyrolactone, and γ-valerolactone(GVL)] mixtures and DMSO solvent was 71.3–80.9% at 140 °C for 40 min. 4 KSCC and Lewis acid Sn-β zeolite as the cocatalyst can dramatically improve the conversion of glucose to HMF. The slightly decrease of the HMF yields were observed from the 2nd cycle to the 4 th cycle, and such phenomenon was because of the deposition of polymer by-products on catalyst surface and the leaching of acid sites from carbon solid acids, revealing that the recyclability of 4 KSCC was fairly good. Li et al. prepared a lignin-based carbon catalyst from Lignin-based activated carbon solid through carbonization and activation at high temperatures to produce HMF from fructose (Li et al. 2020). HMF yield was 75.7% in the DMSO medium, and the yield was 70% after 5 runs, which exhibited good catalytic performance. The heterogeneous catalysts for HMF synthesis from fructose are shown in Table 1.

**Table 1:** Heterogenous catalysts for HMF synthesis from fructose

Entry	Catalysts	Solvents	Reaction conditions	Yield/%	References
1	Ag_3_PW_12_O_40_	H_2_O/MIBK	120 °C, 1 h	77.7	Fan et al. (2011)
2	SO_4_^2−^/TiO_2_	H_2_O/DMSO	150 °C, 6 h	74.7	Tomer and Biswas (2020)
3	SO_4_^2−^/ZrO_2_	[BMIM]Cl	100 °C, 0.5 h	88.4	Qi et al. (2011)
4	SO_4_^2−^/ZrO_2_	DMSO/Acetone	180 °C, 20 min	72.8	Qi et al. (2009a)
5	Si/Al	H_2_O/MIBK	165 °C, 1.5 h	74.0	Moreau et al. (1996)
6	TiO_2_	H_2_O/DMSO	120 °C, 5 min	54.1	Dutta et al. (2011)
7	SBC^a^	THF/DMSO	140 °C, 1 h	92.1	Shen et al. (2016)
8	CSZA-3	DMSO	130 °C, 4 h	56.6	Yan et al. (2009)
9	H_14_[NaP_5_W_30_O_110_]	DMSO	140 °C, 10 min	97.0	Pardo Cuervo et al. (2020)
10	Amberlyst-15/HT	DMA^b^	100 °C, 3 h	76.0	Takagaki et al. (2009)
11	Amberlyst-15	[BMIM]BF_4_/DMSO	80 °C, 32 h	75.0	Lansalot-Matras and Moreau (2003)
12	Amberlyst-15	H_2_O	120 °C, 24 h	15.0	Son et al. (2012)
13	AgFe_2_O_4_@g-C_3_N_4_@SO_3_H	DMSO	90 °C, 1 h	91.0	Niakan et al. (2025)
14	Fe-SC_PSV_	DMSO	120 °C, 0.15 h	80.0	Yang et al. (2025)

^a^*SBC* sulfonated bamboo-derived carbon

^b^DMA, N, N-dimethylacetamide

### The synthesis of HMF from glucose

**Fig. 6:**
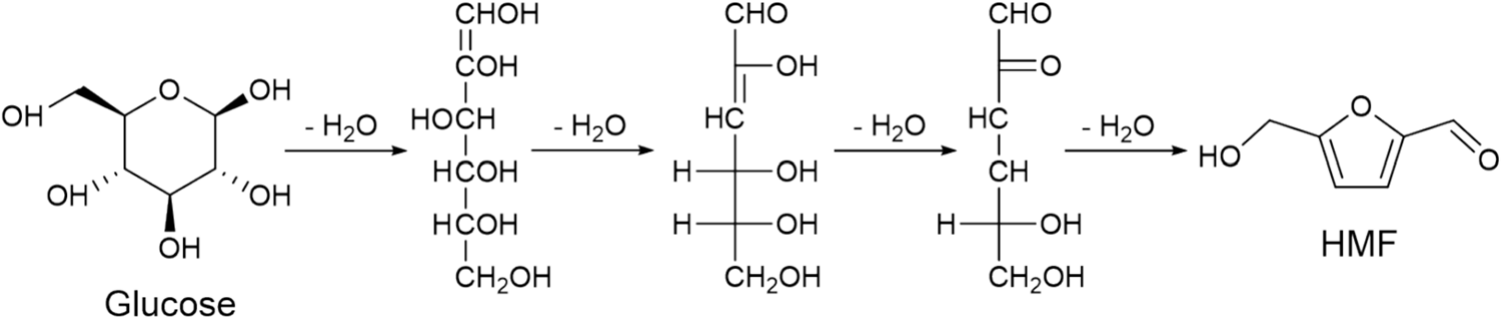
Reaction mechanism of glucose-catalyzed dehydration to prepare HMF (Kuster 1990)

Glucose is readily available from various sources and is inexpensive, making it a promising and economical biomass raw material. HMF yield from glucose usually is higher than that of fructose under the same condition, which is attributed to that the α−1,4-glucosidic bond in glucose is not easily broken, and the pyranoside ring hinders dehydration (Zhang et al. 2017a). Therefore, researchers have been devoted to the development of bifunctional catalysts, which include the Bronsted and Lewis acid sites. Solid acid catalyst systems usually used to prepare HMF from glucose mainly include metal oxides, solid superacids, supported HPAs, zeolites, and metal–organic frameworks (MOFs). The reaction mechanism for glucose dehydration for HMF synthesis is shown in Fig. 6 (Kuster 1990).

Metal oxides with high catalytic activity have many applications in the conversion of biomass. Qi et al. employed ZrO_2_ for HMF synthesis from glucose in [HexylMIM]Cl, and the HMF yield was only 7.0% at 200 °C for 10 min (Qi et al. 2012). With the introduction of H_2_O forming the mixed solvent with [HexylMIM]Cl, the yield of 5-HMF increased to 53.0% at 200 °C for 10 min. This indicated that H_2_O and ionic liquid have a synergistic effect on the catalytic conversion of glucose to HMF. Catalyst recyclability tests revealed a retained HMF yield of 47% at the fifth cycle. Jing et al. studied the HMF synthesis from glucose in H_2_O/THF solvent with 20 wt% NaCl, and 87.8% of HMF yield was obtained (Jing et al. 2018). When CO_2_ was introduced into the reaction system, and HMF yield was increased from 35.7 to 44.2% for 50 min and further increased to 53.8% reacting for 120 min. Furthermore, solid catalyst was recycled for at least 4 times and activated through calcination without any structural transformation. Marianou et al. employed SnO_2_/γ-Al_2_O_3_ as the catalyst to perform the HMF synthesis from glucose with 27.5% HMF yield in H_2_O/DMSO mixed solvent at 150 °C for 1 h (Marianou et al. 2018). After the first reaction, residual coke, humin, and unreacted compounds loaded on the catalyst were calcined at low temperatures. The regenerated catalyst still showed high activity, which was close to the result obtained by the fresh catalyst. Silahua-Pavón et al. synthesized TiO_2_-ZrO_2_ with various additives (CH_3_COOH, HNO_3_, NaOH, and NH_4_OH) through the sol–gel method, among which TiZr5050-C_2_H_4_O_2_ represented good catalytic performance on the glucose dehydration to HMF (Silahua-Pavón et al. 2019). 76% HMF yield was in H_2_O/THF biphasic system at 175 °C and 30 bar of Ar for 1 h. However, HMF yield decreased to 60% after three runs, which was due to the stability and carbon deposits on the catalyst surface.

Yan et al. prepared SO_4_^2−^/ZrO_2_ and SO_4_^2−^/ZrO_2_-Al_2_O_3_ catalysts through impregnation, and 47.6% of HMF yield from glucose can be reached using SO_4_^2−^/ZrO_2_-Al_2_O_3_ (Zr/Al = 1/1) at 130 °C for 4 h (Yan et al. 2009). They found that it was favorable to form HMF using the catalyst with higher acidity and moderate basicity. Osatiashtiani et al. prepared SO_4_^2−^/ZrO_2_ catalysts with tuneable acid sites to convert glucose to HMF through the one-pot method (Osatiashtiani et al. 2014). They found that SO_4_^2−^/ZrO_2_ with appropriate degrees of surface sulfation will promote HMF synthesis from glucose. The monoclinic ZrO_2_ was formed without sulfation and had Lewis acid and base sites, which can enhance the process of glucose isomerization to fructose but be adverse to HMF synthesis from fructose. SO_4_^2−^/ZrO_2_ was prepared after sulfuric acid impregnation and calcination, which stabilized tetragonal ZrO_2,_ introduced Brønsted acid sites, and promoted HMF synthesis. SO_4_^2−^/ZrO_2_ with submonolayer sulfate coverages formed through 0.3 M sulfuric acid impregnation. Hoang et al. employed SO_4_^2–^/ZrO_2_ as the catalyst to catalyze glucose dehydration in the NaCl–THF/H_2_O biphasic solvent system (Hoang and Cuong 2021). The biphasic solvent can improve the selectivity of HMF, and the highest HMF yield was achieved at 160 °C for 2 h. Meanwhile, another bio-based platform chemical levulinic acid was obtained with a yield of 29.68%. Tomer and Biswas prepared SO_4_^2−^/TiO_2_ through sol–gel hydrolysis and impregnation to prepare HMF from glucose, and 37% HMF yield was obtained catalyzed by 0.5 M SO_4_^2−^/TiO_2_ in DMSO solvent at 150 °C for 6 h (Tomer and Biswas 2020). This catalyst contained the proportion of Lewis and Brønsted acid sites, which can convert glucose to HMF efficiently. They found that the process of glucose dehydration to HMF was a second-order reaction.

HPAs are also applied in the catalytic glucose conversion, while they usually exist in the form of insoluble solids. Fan et al. synthesized insoluble HPA salt Ag_3_PW_12_O_40_ to prepare HMF from glucose in H_2_O/MIBK biphasic solvent. The 76.3% HMF yield with 85.3% selectivity was reached at 130 °C for 4 h, and Ag_3_PW_12_O_40_ can exhibit excellent catalytic performance in high-concentration raw material. Pardo Cuervo et al. employed commercial Preyssler HPA (H_14_[NaP_5_W_30_O_110_]) to prepare HMF from glucose in DMSO solvent (Pardo Cuervo et al. 2020). The 23.0% HMF yield with 91% glucose conversion can be obtained at 140 °C for 9 h, which is much lower than that of fructose as the raw material. This catalyst can also catalyze xylose dehydration to form furfural with a 73% yield in the DMSO solvent. The Preyssler HPA was recovered through the easy and efficient methodology and was reused at least six cycles without significant catalytic activity decrease. Nogueira et al. prepared several heterogeneous catalysts with HPAs (HPW and HPMo) and supports (Nb_2_O_5_ and Al_2_O_3_) at different calcination temperatures for HMF synthesis from glucose, and HPW/Nb_2_O_5_ calcined at 300 °C was selected (Siqueira Mancilha Nogueira et al. 2020). They employed Taguchi’s L16 experimental design to obtain 40.8% HMF yield from glucose at 160 °C for 30 min in H_2_O/acetone mixed solvent. HMF yield remained about 40% using this catalyst during four runs, exhibiting high stability and good recyclability. Zhao et al. prepared several bifunctional ionic hybrid catalysts with [MimAM]Br ionic liquid and HPW (Zhao et al. 2018b). HMF yield from glucose dehydration catalyzed by the insoluble [MimAM]H_2_PW_12_O_40_ was 53.9% at 160 °C for 7.5 h in THF/H_2_O-NaCl solvent, which is a little higher than that catalyzed by soluble HPW. This indicated that [MimAM]H_2_PW_12_O_40_ with the synergistic effect of dual acidic properties showed good performance. When the reaction was conducted in DMSO, HMF yield reached 83.2%. However, [MimAM]H_2_PW_12_O_40_ was soluble in DMSO and its high boiling point was unfavorable to the separation and purification of HMF. Chang et al. synthesized heterogenous SiO_2_-ATS-PTA (ATS, 3-aminopropyltrimethylhydrosilane and PTA, phosphotungstic acid) as the catalyst to convert glucose to HMF (Chang et al. 2022). The highest yield of HMF was 74.67% in [EMIM]Cl at 140 °C for 180 min. After repeated use of the catalyst for three runs, the HMF yield decreased slightly. This result indicated that the SiO_2_-ATS-PTA catalyst showed good catalytic activity and stability for HMF synthesis from glucose in [EMIM]Cl.

**Fig. 7:**
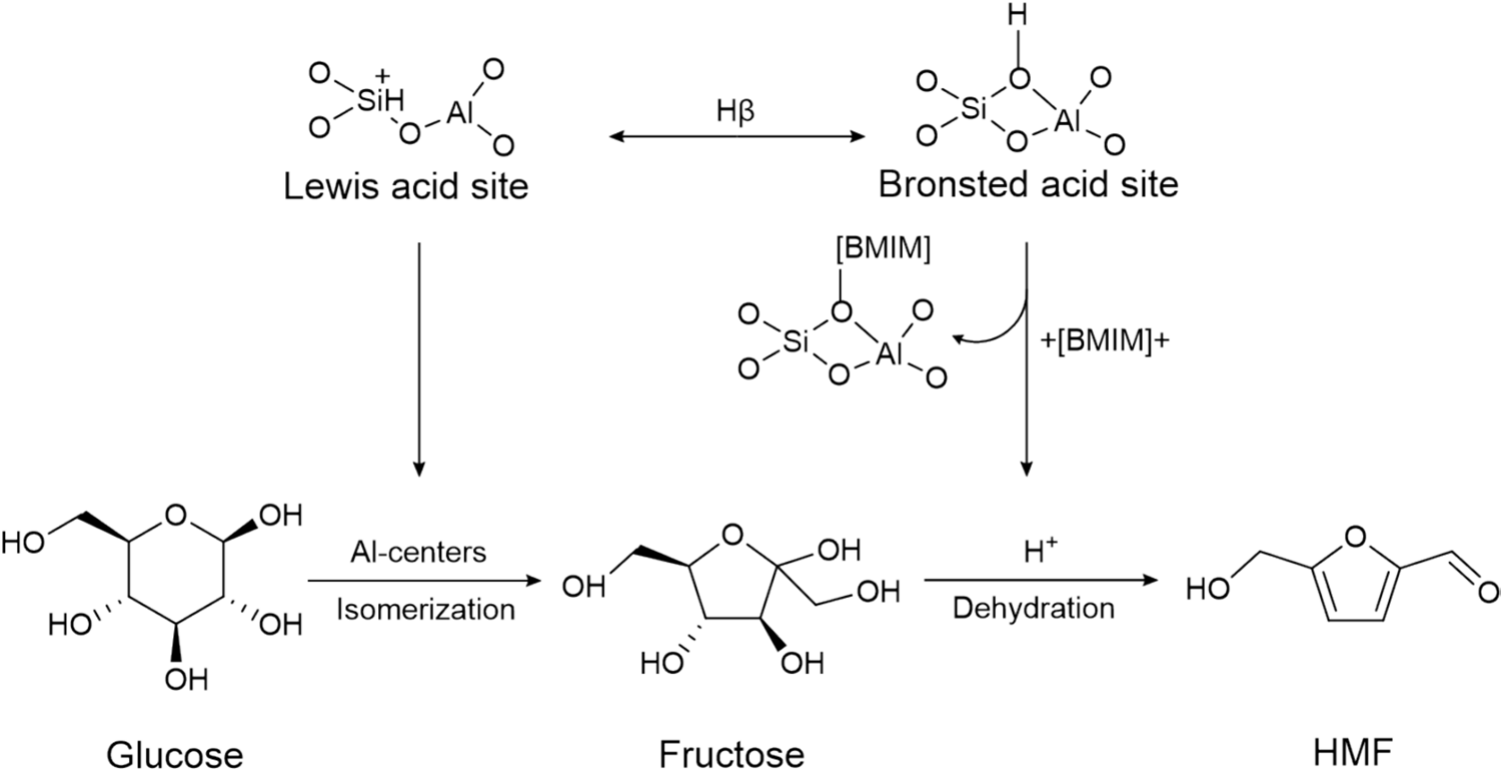
The catalytic mechanism of Hβ zeolite with Lewis and Brønsted acid sites for HMF synthesis from glucose (Hu et al. 2014)

Zeolites were also widely used for the conversion of glucose due to their uniform structure, high thermal stability, and adjustable acidity (Kruger et al. 2013; Xu et al. 2023). Usually, zeolites contain both Lewis and Brønsted acid sites, which play a vital role in the catalytic process. In the process, Lewis acid sites enhance glucose isomerization to fructose, and Brønsted acid sites promote fructose dehydration to HMF. Therefore, many researchers have been focusing on the acidity and acid site regulation of zeolites to enhance glucose conversion and its selectivity to HMF. Dealumination or desilication was employed to regulate acidity, pore structure, thermal stability, and catalytic performance of zeolites, which can be achieved by mineral acid treatment or heteroatom (Sn, Zr, Ti) insertion in zeolite framework (Moliner 2014). Candu et al. prepared mesoporous Nb-Beta zeolite from dealuminized H-Beta with Nb ethoxide impregnation and employed this catalyst to convert glucose to HMF (Candu et al. 2019). The highest selectivity of HMF was 84.3% at 180 °C for 12 h in H_2_O/MIBK-NaCl solvent. Nb(V)O–H in Nb-Beta zeolite showed moderate strength Brønsted acidity, which was beneficial to the stability of the Nb-Beta framework. This catalyst was recovered by calcination and exhibited good catalytic performance during four runs. Saenluang et al. synthesized Sn-incorporated Beta nanocrystals through an *in-situ* hydrothermal process with bifunctional catalytic activity (Lewis and Brønsted acid) for HMF synthesis from glucose (Saenluang et al. 2020). The introduction of Sn decreased the crystallinity of Beta zeolite, which can be inferred from their XRD patterns. Sn as Lewis acid sites can promote the process of glucose isomerization to fructose. The highest yield of HMF from glucose was 42.0% at 120 °C for 24 h in dioxane/H_2_O solvent. Zhang et al. synthesized several Sn-Al-Beta catalysts with bifunctionality from commercial H-Beta zeolite through the ion exchange method (Zhang et al. 2023). Among the catalysts, Sn-Al-Beta-4–8 showed excellent catalytic activity for HMF synthesis from glucose, and the highest HMF yield was 54% at 120 °C for 2 h in [C4 mim]Cl. This was due to a balance between Lewis and Brønsted acid sites for HMF synthesis. After five cycles, the yield of HMF decreased to 41%, which was attributed to the attachment of insoluble byproducts on the catalyst surface. Besides, this catalyst exhibited good catalytic performance on other carbohydrates (sucrose, starch, cellobiose, and cellulose) for HMF production, and the yields were 59%, 38%, 47%, and 41%, respectively. The catalytic mechanism of the zeolite for the conversion of glucose into HMF is shown in Fig. 7 (Hu et al. 2014).

MOFs are highly crystalline porous materials with an ordered structure, which are formed by the self-assembling of metal clusters or metal ions with multidentate ligands. Due to modifiable microarchitecture, tunable pore size, and high surface area, MOFs exhibit great potential as a new type of catalyst and are widely used in biomass conversion (Fang et al. 2020; Xu et al. 2022). Tangsermvit et al. synthesized MOF catalysts for the preparation of HMF (Tangsermvit et al. 2021). The HMF yield from glucose increased from 2.70 to 14.06% catalyzed by UiO-66 and sulfonated UiO-66 (U66S). A1^3+^ sites were incorporated on Brønsted acid U66S, forming U66SA with Brønsted and Lewis acid sites. The HMF yield from glucose increased to 63.00% by using U66SA, which was due to the cooperative effect and high acid density from Brønsted and Lewis acid sites. ZIF-8 MOF prepared by hydrothermal synthesis with a high surface area was used for HMF synthesis from glucose (Oozeerally et al. 2019). Due to lacking strong Brønsted acidity in ZIF-8 MOF, the reaction was carried out at pH = 1.0 to improve HMF yield. However, the activity of ZIF-8 decreased dramatically in a flow reactor at 100 °C for 3 h. MOFs were supported on carbon nitride materials with high stability, low cost, and excellent catalytic activity for HMF synthesis (Zhang et al. 2020b). Bifunctional active sites were introduced in UiO-66/C_3_N_4_@PDA, which resulted in 54.9% HMF yield from glucose in isopropanol/DMSO solvent. This catalyst can convert other carbohydrates efficiently into HMF and also exhibits superior recyclability for HMF synthesis from glucose. Due to the synergistic effects of two metals in MOFs, mixed-metal MOFs exhibited better catalytic performance than single-metal MOFs. Lu et al. prepared MIL-101(Cr, Fe) and Fe/MIL-101(Cr) through different synthetic methods for HMF synthesis (Lu et al. 2023). The HMF yield from glucose was 11.1% catalyzed by MIL-101(Cr) at 150 °C in DMSO/water solvent, while the yield increased to 48.7% using Fe/MIL-101(Cr) as the catalyst at the same conditions. The highest HMF yield from glucose was 55.8% catalyzed by Fe/MIL-101(Cr) at 170 °C for 2 h in the DMSO/water solvent, and this catalyst remained the main catalytic activity desired after five runs. Table 2 summarizes the recent progress over heterogeneous catalysts for HMF synthesis from glucose.

**Table 2:** Heterogeneous catalysts for HMF synthesis from glucose

Entry	Catalysts	Solvents	Reaction conditions	Yield/%	References
1	SnO_2_/γ-Al_2_O_3_	H_2_O/DMSO	150 °C, 1 h	27.5	Marianou et al. (2018)
2	Sn/Al_2_O_3_	[EMIM]Br	140 °C, 3 h	50.8	Bai et al. (2020)
3	TiO_2_ NPs	H_2_O/Butanol	175 °C, 3 h	81.0	Atanda et al. (2015a)
4	SiSnPO	H_2_O/DMSO	180 °C, 1.5 h	70.3	Zhang et al. (2021)
5	SO_4_^2−^/TiO_2_	H_2_O/DMSO	150 °C, 6 h	37.0	Tomer and Biswas (2020)
6	H_14_[NaP_5_W_30_O_110_]	DMSO	140 °C, 9 h	23.0	Pardo Cuervo et al. (2020)
7	Ag_3_PW_12_O_40_	H_2_O/MIBK	130 °C, 4 h	76.3	Fan et al. (2011)
8	[Sn,Al]-β	DMSO	160 °C, 4 h	37.5	Qing et al. (2022)
9	SAPO-34/5 A	H_2_O	190 °C, 3 h	20.0	Romo et al. (2018)
10	Sn/SAPO-34	35wt% NaCl- H_2_O/THF	150 °C, 1.5 h	64.4	Song et al. (2021)
11	2SZ@SBA-15-SO_3_H-NH_2_	H_2_O/DMSO	120 °C, 3 h	68.6	Zhang et al. (2017b)
12	Fe_3_O_4_@SiO_2_-SO_3_H	H_2_O/MIBK	140 °C, 24 h	70.0	Elsayed et al. (2018)
13	U66SA	H_2_O/DMSO	120 °C, 24 h	63.0	Tangsermvit et al. (2021)
14	MIL-101 Cr-SO_3_H	H_2_O/THF	130 °C, 24 h	29.0	Herbst and Janiak (2016)
15	UiO-66-type MOFs	DMSO	140 °C, 12 h	49.7	Zhang et al. (2019)
16	UiO-66-NH_2_-SO_3_H/C_3_N_4_@PDA	IPA/DMSO	120 °C, 6 h	54.9	Zhang et al. (2020b)
17	SAPO_34	γ-Valerolactone/water	170 °C, 1.5 h	92	Rostami et al. (2025)
18	t-SiO_2_@B@A	H_2_O	120 °C, 6 h	69.7	Kang et al. (2025)

### The synthesis of HMF from cellulose and raw biomass

As the most abundant natural non-food polysaccharide, cellulose comes from various sources, including agricultural waste, livestock and poultry manure, woody biomass, and marine biomass. The preparation of HMF from cellulose and raw biomass directly can effectively reduce environmental burden and production costs. This reaction was carried out as follows: cellulose → glucose → fructose → HMF. At present, much research has focused on HMF synthesis from cellulose and raw biomass with various heterogeneous catalysts.

Zeolites with heteroatom insertion in their framework were also employed in the conversion of cellulose. Sezgin et al. (2019) prepared Cr -Beta and Cr-USY for HMF synthesis from cellulose, and 34.1% and 34.3% of HMF yield were achieved with Cr-Beta (Si/Al = 28) and Cr-USY (Si/Al = 6) in [BMIM]Cl at 130 °C for 1 h, respectively. Hf/ZSM-5 zeolite was prepared and used as the catalyst for HMF synthesis from cellulose, and 67.5% HMF yield was reached in H_2_O/THF/NaCl at 190 °C for 2 h (Wu et al. 2021). The introduction of metal chloride can enhance HMF yield due to the increase of Lewis acidic sites (Binder and Raines 2009). Then, Xu et al. added metal chloride into the reaction system and investigated its effect on HMF synthesis (Xu et al. 2024). They found that the highest HMF yield was 70.95% with AlCl_3_ and Hf/ZSM-5 as the catalyst in the optimal reaction conditions. Rapado et al. (2024) employed the combination of heterogenous β-zeolite and homogenous HCl as the catalyst to prepare HMF from microcrystalline cellulose under mild conditions.

Metal oxides were also used for the preparation of HMF synthesis from cellulose. Jing et al. employed ZrO_2_ and TiO_2_ as the catalyst with in-situ carbonic acid from CO_2_ to improve the conversion of cellulose to HMF (Jing et al. 2018). HMF yield was 48.4% in H_2_O/THF system at 200 °C for 3 h. The solid catalyst showed excellent catalytic activity in the presence of CO_2_ during the four runs. The HMF yield increased from 47.2 to 54.7%, which may be due to the partial accumulation of cellulose. Mo et al. used waste cotton stalks as raw materials for catalytic degradation for HMF synthesis. SO_4_^2−^/ZrO_2_ was employed as an effective catalyst for the degradation of cotton stalks, and the yield of HMF achieved 27.2% at 230 °C for 75 min. The recovered SO_4_^2−^/ZrO_2_ was reused as catalyst for the preparation of HMF from cycle 1 to cycle 4 under the optimized reaction conditions. The yields of HMF (from cycle 1 to cycle 4) are 27.2%, 26.2%, 25.9% and 25.4%, respectively (Mo et al. 2017).

HPAs were used as the catalysts with high Brønsted acid strength for HMF synthesis from cellulose, while their solubility in solvents limited their application. Zhang et al. prepared several HPW-based catalysts with choline chloride (Ch) and HPW to prepare HMF from cellulose in a one-pot method (De Andrades et al. 2024). 49.1% HMF yield from cellulose was achieved with HPW in H_2_O/MIBK biphasic system at 140 °C for 8 h. Among the prepared heterogeneous catalysts, Ch_2_HPW_12_O_40_ exhibited the highest catalyst active and achieved 75.0% HMF yield from cellulose, which was comparable to that of HPW. Moreover, the selectivity of Ch_2_HPW_12_O_40_ was larger than HPW. Ch_2_HPW_12_O_40_ was insoluble in cool water, while it became soluble in water with the reaction temperature increasing to 60 °C. This can be conducive to the separation from the reaction mixture and improve the contact with the reactant during the reaction. After ten runs, the recovery of Ch_2_HPW_12_O_40_ was about 94.3% and the HMF yield remained high level. The temperature-responsive Ch_n_H_5-n_CeW_12_O_40_ was also prepared for HMF synthesis as follows: the synthesis of H_5_CeW_12_O_40_ from HPW, CeCl_3_·7H_2_O and Na_2_WO_4_·H_2_O, and the synthesis of Ch_n_H_5-n_CeW_12_O_40_ from ChCl solution and H_5_CeW_12_O_40_ (Lai et al. 2020). Among the HPA-based catalysts, the highest HMF yield from cellulose was reached catalyzed by ChH_4_CeW_12_O_40_ (> 80 °C, soluble; < 80 °C, insoluble) in H_2_O/DMSO/MIBK system at 140 ^o^C for 6 h. This was due to the synergistic effect of Brønsted-Lewis acidity and homogeneous catalysis of the temperature-responsive ChH_4_CeW_12_O_40_. The feasibility of this catalyst for various polysaccharides (fructose, glucose, cellobiose, and starch) was studied, and their HMF yields were 81.3%, 71.2%, 75.8%, 70.2%, and 68.7%, respectively. Nogueira et al. synthesized heterogenous HPW/Nb_2_O_5_ for t HMF production from cellulose (Nogueira et al. 2020). The HMF yield from cellulose achieved 20.6% in acetone/H_2_O solvent at 200 °C for 2 h. The HMF yield from various raw biomass (microcrystalline cellulose, commercial eucalyptus cellulose pulp, and brewer’s spent grain cellulose pulp) was 11.7–18.1%.

MOFs were used in catalytic reactions due to their adjustable acidity and acid sites through coordinate acid and base functional ligands. Usually, the introduction of the functional group into MOFs can enhance their catalytic performance. Zhao et al. prepared PHs-SO_3_H@UiO-66-NH_2_ with hierarchical porous structure and multifunctional active sites by chemically grafting UiO-66(Hf)-type MOFs onto sulfonated Poly-Pickering HIPEs (PHs-SO_3_H) (Zhao et al. 2018a). This catalyst was employed for the conversion of cellulose to HMF, and the highest HMF yield was 49.6% at 120 °C for 60 min. Wei et al. synthesized dual acid–base bifunctional catalyst from Pickering HIPEs as template and MOFs (UiO-66-SO_3_H and UiO-66-NH_2_)/Tween 85 as co-stabilizers for HMF synthesis from cellulose (Wei et al. 2022). Among the prepared catalysts, Poly-P12 with great recyclability exhibited the best catalytic performance, and the highest HMF yield was 40.5% at 130 °C for 3 h in [EMIM]Cl. Lu et al. prepared mixed-metal Sn/UiO-66-SO_3_H catalysts to produce HMF from carbohydrates, and the synergistic effect of Zr^4+^ and Sn^4+^ can efficiently enhance the catalytic performance of Sn/UiO-66-SO_3_H (Lu et al. 2024). The reactions were carried out in green deep eutectic solvent betaine-malic acid–water/MIBK biphasic system, and 41.7% of HMF yield from cellulose was obtained using 24-Sn/UiO-66-SO_3_H as the catalyst. Table 3 summarizes the recent progress o heterogeneous catalysts for HMF synthesis from cellulose and raw biomass.

**Table 3:** Heterogeneous catalysts for HMF synthesis from cellulose and raw biomass

Entry	Feedstocks	Catalysts	Solvents	Reaction conditions	Yield/%	References
1	Remnant algal	ZSM-5	H_2_O/THF/NaCl	170 °C, 1 h	34.4	Rihko-Struckmann et al. (2020)
2	Cellulose	Hf/ZSM-5	H_2_O/THF/NaCl	190 °C, 2 h	67.5	Wu et al. (2021)
3	Cellulose	Cr-USY(Si/Al = 6)	BMIMCl	130 °C, 2 h	34.3	Sezgin et al. (2019)
4	Cellulose	Ch_2_HPW_12_O_40_	H_2_O/MIBK	140 °C, 8 h	75.0	De Andrades et al. (2024)
5	Cellulose	ZrO_2_, TiO_2_	H_2_O/THF	200 °C, 3 h	48.4	Jing et al. (2018)
6	Cotton stalk	SO_4_^2−^/ZrO_2_	H_2_O	230 °C, 75 min	27.2	Rapado et al. (2024)
7	Cellulose	ChH_4_CeW_12_O_40_	H_2_O/DMSO/MIBK	140 °C, 6 h	67.5	Lai et al. (2020)
8	Cellulose	HPW/Nb_2_O_5_	Acetone/H_2_O	200 °C, 0.5 h	20.6	Nogueira et al. (2020)
9	Cellulose	(30%)SO_4_^2−^/HfO_2_	H_2_O/THF	220 °C, 5 h	54.0	Shi et al. (2023)
10	Cellulose	P-TiO_2_	H_2_O/MIBK/NMP	180 °C, 1.75 h	53.0	Atanda et al. (2015b)
11	Carboxymethyl cellulose	MIL-53(Al)	H_2_O	200 °C, 4 h	40.3	Zi et al. (2015)
12	Holocellulose	H_2_N-UiO-66-SO_3_	H_2_O/GVL	190 °C, 6 h	39.1	Fan et al. (2023)

## The synthesis of FDCA from HMF

**Fig. 8:**
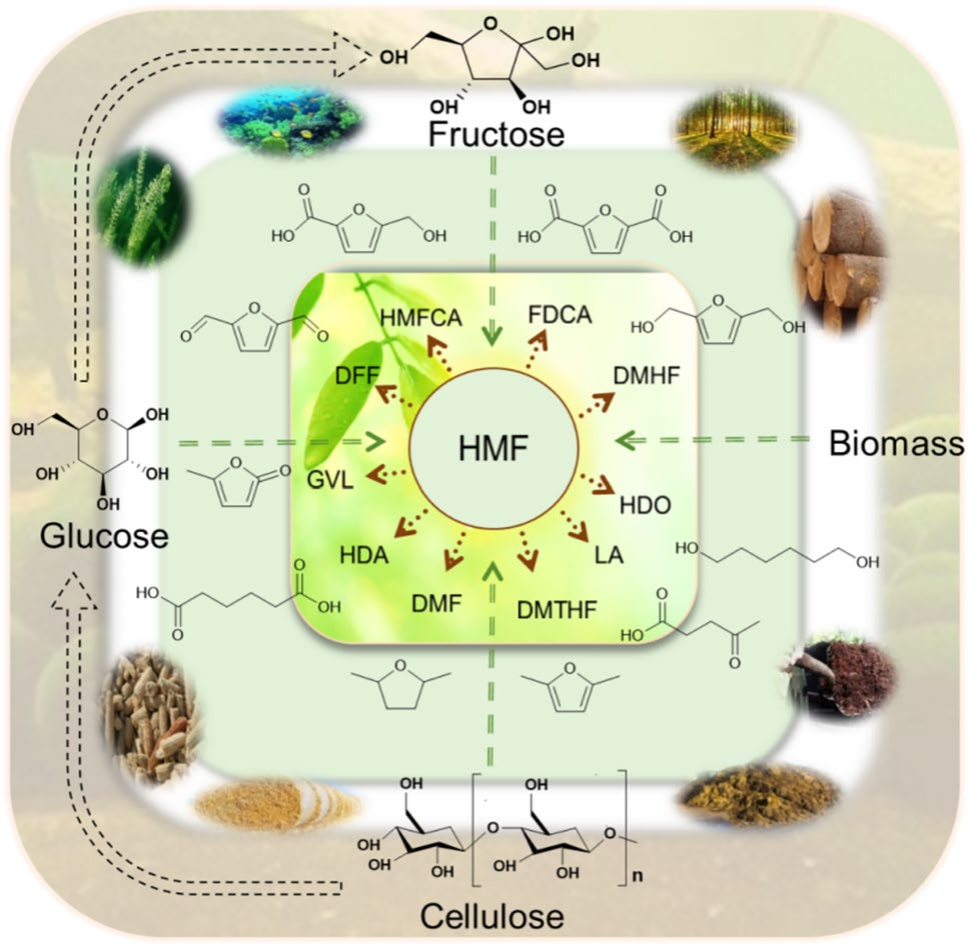
HMF synthesis from biomass and its derivatives

**Fig. 9:**
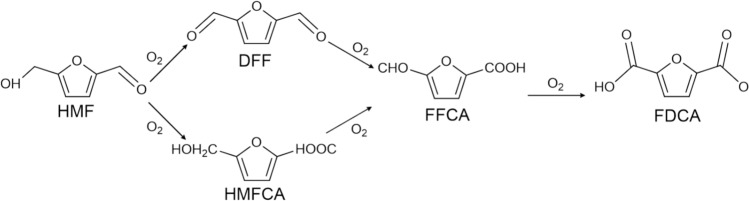
Possible routes of FDCA production from HMF (Saha et al. 2013)

HMF served as a bridge connecting biomass resources and petrochemicals as a promising platform compound. It can prepare 5-hydroxymethyl-2-furancarboxylic acid (HMFCA), FDCA, 2,2-diformylfuran (DFF), GVL, LA, DMF, and other derivatives through oxidation, reduction, hydrogenation, and condensation as shown in Fig. 8. They are widely used in industries such as plastics, chemicals, petroleum additives, food, etc. (Yang et al. 2020). Among these derivatives, FDCA is one of the important downstream products through selective oxidation of HMF. FDCA contains a furan ring and two more active aldehyde groups, which can be employed to synthesize a large number of high value-added compounds and new bio-based polymer materials through chemical reactions such as hydrogenation, oxidation, polymerization, and hydrolysis (Rao et al. 2021). FDCA is an important degradable bio-material monomer, which can be used as a substitute chemical for petroleum-based terephthalic acid (PTA) to prepare renewable high-performance polymers. The as-prepared CoOx-MC exhibited superior performance in the selective oxidation of 5-HMF to FDCA using O_2_ as the oxidant in aqueous medium under mild conditions. Recycling tests demonstrated no significant loss of catalytic activity after three consecutive cycles. Furthermore, characterization of the used CoOx-MC revealed nearly identical physicochemical properties compared to the fresh catalyst (Liu et al. 2020). Polyethylene furanoate (PEF) prepared from FDCA can be used as a substitute for polyethylene terephthalate (PET) from PTA (Guan et al. 2021; Wang et al. 2020). PET is widely used in industries such as electrical appliances, automobiles, machinery, plastics, and so on (Peng et al. 2023). It exhibited excellent properties, such as friction resistance, fatigue resistance, creep resistance, and dimensional stability, but it also has a long production cycle, poor impact performance, and slow crystallization rate (Dhaka et al. 2022). Compared with PET, PEF has higher mechanical strength, superior barrier performance, better thermal performance, and better recyclability and renewability (Hwang et al. 2020). Thus, PEF has extensive application potential in the field of high-barrier packaging materials, high-performance fibers, and engineering plastics. At present, many researchers have been focusing on the production of FDCA from HMF, and the preparation methods mainly include direct oxidation, electrocatalytic oxidation, enzymatic catalysis, metal catalysis, etc. The preparation route of FDCA from HMF is shown in Fig. 9.

Direct oxidation method is to oxidize the hydroxyl and aldehyde groups of HMF molecule to carboxyl groups and produce FDCA using oxidizing agents. According to the oxidant types, the selective oxidation reactions of FDCA on the oxidation route can be divided into traditional inorganic oxidants and green oxidants. Zhao et al. employed ruthenium-based materials Ru/C as catalysts and H_2_O_2_ as the oxidant to convert HMF into FDCA in water or organic solvents (Zhao et al. 2020a, b). This method uses fewer harmful chemicals and is more environmentally friendly compared to other methods. However, it is still necessary to improve catalytic efficiency and reduce production costs.

Electrocatalytic oxidation method uses electrode potential as the main driving force, and HMF undergoes an oxidation reaction at the anode to form FDCA. Wang et al. prepared the NiCoP electrode as an electrocatalyst and achieved the electro-oxidation reaction of HMF to FDCA through surface reconstruction of the catalyst (Wang et al. 2021). The route of FDCA from HMF is as follows: HMFCA from HMF, 5-formyl-2-furancarboxylic acid (FFCA) from HMFCA, and the formation of FDCA. This method is environmentally friendly and performed at room temperature, but there are also challenges such as improving reaction efficiency, reducing costs, and optimizing reaction conditions.

Enzymatic method employed biocatalysts to promote chemical reactions of FDCA from HMF. Milic et al. reported the concept of a one-pot biocatalytic cascade for the preparation of FDCA using HMF (Milić et al. 2022). HMF is oxidized to DFF using galactose oxidase in a biphasic or micro aqueous system, which is then oxidized to FDCA by lipase-mediated peroxidation. The enzymatic method can be carried out at mild operating conditions with environmental friendliness and high selectivity, but it also has some problems such as enzyme stability, substrate conversion, and product inhibition.

Metal catalysts for FDCA from HMF include noble and non-noble metal catalysts. Zhong et al. successfully prepared an efficient and stable catalyst 1.5Pt_1_Au_4_/N-HNT with APTES-functionalized halloysite nanotubes (N-HNT) of Pt-Au alloy nanoparticles for the conversion of HMF to FDCA (Zhong et al. 2022). HMF conversion and FDCA selectivity were 100.0% and 99.0% under optimal conditions. Kar et al. used a Ru-based catalyst with alkaline water as the oxidant for the oxidation of HMF to FDCA directly (Kar et al. 2022). The catalytic mechanism showed that the initial hydride is transferred to the catalyst ligand skeleton from the substrate to produce dearomatized complexes, which then achieved a 95.0% yield of FDCA through catalytic oxidation. The noble metal catalysts have higher selectivity and are more conducive to the yield of FDCA from HMF compared with non-noble metal catalysts. Liu et al. employed O_2_ as an oxidant and CoO_x_-MC as the catalyst to selectively oxidize HMF to FDCA in water (Liu et al. 2020). The conversion of HMF was 98.3%, and the yield of FDCA was 95.3% at 80 °C for 30 h. As one of the most promising non-noble metal catalysts, Co can achieve high conversion and selectivity even under very mild oxidation conditions. Non-noble metal catalysts have significant advantages in cost and environmental friendliness but still exist challenges in catalytic efficiency, stability, and production costs. Therefore, it is quite necessary to develop low-cost metal non-noble catalysts with high catalytic performance.

## Conclusions and perspectives

This review summarizes an overview of recent advancements in research and the prospective applications of homogeneous catalysts in synthesis of high-value HMF and its oxidation derivatives. Meanwhile, the review systematically ‌analyzes‌ the reaction pathways and catalytic mechanisms for the synthesis of HMF using ‌inorganic acids, organic acids, metal salts, and ionic liquids‌, and ‌evaluates‌ the cycle life of these catalysts. The pathways for synthesizing HMF from ‌glucose, fructose, and biomass‌ under varying reaction conditions ‌are compared and analyzed‌. FDCA‌, a critical biodegradable biopolymer precursor, is then introduced. Furthermore, we ‌examine‌ the preparation methods for FDCA synthesis from biomass, including ‌direct oxidation, electrocatalytic oxidation, and enzymatic methods‌.

Although HMF synthesis has been extensively studied over decades, persistent challenges remain in economic viability, environmental sustainability, and conversion efficiency across various feedstocks. To address these limitations, future research should prioritize expanding substrate utilization to cost-effective natural polysaccharides, including agricultural residues like corn stover, wheat straw, and other non-edible lignocellulosic biomass. Concurrently, advancements in three key areas are required: (1) design of high-performance multiphase catalytic systems, (2) development of novel green solvents, and (3) innovation in energy-efficient separation technologies. The realization of economically feasible, large-scale HMF production would catalyze transformative developments in HMF-derived products and significantly propel progress in biomass valorization technologies.

## Data Availability

Not applicable.
